# Experimental phantom evaluation to identify robust positron emission tomography (PET) radiomic features

**DOI:** 10.1186/s40658-021-00390-7

**Published:** 2021-06-12

**Authors:** Montserrat Carles, Tobias Fechter, Luis Martí-Bonmatí, Dimos Baltas, Michael Mix

**Affiliations:** 1 Division of Medical Physics, Department of Radiation Oncology, Medical Center - University of Freiburg, Faculty of Medicine, University of Freiburg, Freiburg im Breisgau, Germany; 2 German Cancer Consortium (DKTK), German Cancer Research Center (DKFZ), Partner Site Freiburg, German Cancer Research Center (DKFZ), Heidelberg, Germany; 3 Biomedical Imaging Research Group (GIBI230-PREBI) and Imaging La Fe node at Distributed Network for Biomedical Imaging (ReDIB) Unique Scientific and Technical Infrastructures (ICTS), La Fe Health Research Institute, Av. Fernando Abril Martorell, 106, 46026 Valencia, Spain; 4 Department of Nuclear Medicine, Medical Center - University of Freiburg, Faculty of Medicine, University of Freiburg, Freiburg im Breisgau, Germany; 5 Department of Medical Imaging and Clinical Oncology, Nuclear Medicine Division, Faculty of Medicine and Health Science, Stellenbosch University, Stellenbosch, South Africa

**Keywords:** Radiomic features, PET, Robustness, Phantoms, Heterogeneity

## Abstract

**Background:**

Radiomics analysis usually involves, especially in multicenter and large hospital studies, different imaging protocols for acquisition, reconstruction, and processing of data. Differences in protocols can lead to differences in the quantification of the biomarker distribution, leading to radiomic feature variability. The aim of our study was to identify those radiomic features robust to the different degrading factors in positron emission tomography (PET) studies. We proposed the use of the standardized measurements of the European Association Research Ltd. (EARL) accreditation to retrospectively identify the radiomic features having low variability to the different systems and reconstruction protocols. In addition, we presented a reproducible procedure to identify PET radiomic features robust to PET/CT imaging metal artifacts. In 27 heterogeneous homemade phantoms for which ground truth was accurately defined by CT segmentation, we evaluated the segmentation accuracy and radiomic feature reliability given by the contrast-oriented algorithm (COA) and the 40% threshold PET segmentation. In the comparison of two data sets, robustness was defined by Wilcoxon rank tests, bias was quantified by Bland–Altman (BA) plot analysis, and strong correlations were identified by Spearman correlation test (r > 0.8 and p satisfied multiple test Bonferroni correction).

**Results:**

Forty-eight radiomic features were robust to system, 22 to resolution, 102 to metal artifacts, and 42 to different PET segmentation tools. Overall, only 4 radiomic features were simultaneously robust to all degrading factors. Although both segmentation approaches significantly underestimated the volume with respect to the ground truth, with relative deviations of −62 ± 36% for COA and −50 ± 44% for 40%, radiomic features derived from the ground truth were strongly correlated and/or robust to 98 radiomic features derived from COA and to 102 from 40%.

**Conclusion:**

In multicenter studies, we recommend the analysis of EARL accreditation measurements in order to retrospectively identify the robust PET radiomic features. Furthermore, 4 radiomic features (area under the curve of the cumulative SUV volume histogram, skewness, kurtosis, and gray-level variance derived from GLRLM after application of an equal probability quantization algorithm on the voxels within lesion) were robust to all degrading factors. In addition, the feasibility of 40% and COA segmentations for their use in radiomics analysis has been demonstrated.

**Supplementary Information:**

The online version contains supplementary material available at 10.1186/s40658-021-00390-7.

## Introduction

Radiomics is defined as the extraction and analysis of a large amount of quantitative image features from standard-of-care images, known as radiomic features (RF). Radiomics studies have four main steps to be considered [16]: image acquisition and reconstruction, volume segmentation and preprocessing, radiomic features extraction, and development and validation of descriptive models. The descriptive models resulted from radiomics analysis are expected to be generalizable. Therefore, the radiomic features involved in the process require a high level of robustness [35].

Quantitative analysis of positron emission tomography (PET) images is considered an established method for diagnosis, staging, and evaluation of tumor response to therapy [32]. Unfortunately, the lower spatial resolution and poor statistics of PET images have a negative impact on the variability of radiomic features, in comparison with other imaging modalities such as computed tomography (CT). In addition, previous studies have reported that PET radiomic feature variability also increased due to the differences in image acquisition parameters, image reconstruction algorithms, and lesion segmentation procedures [24, 34]. Consequently, PET radiomic feature variability should be properly addressed in order to avoid misinterpretation of the developed descriptive models when using PET images.

Retrospective analysis of clinical data usually involves, especially in multivendor and multicenter studies, different imaging systems, system updates, reconstruction protocols, and segmentation methods. A solution to minimize radiomic feature variability due to this pipeline heterogeneity could be to restrict the data to those patients following exactly the same protocols (in both acquisition and image processing), as in clinical trials. However, these constraints will reduce the number of patients and would compromise the generalizability of the model. In order to avoid the subsample size bias, an alternative approach is to identify those radiomic features robust to the different vendors, protocols, and methods in the pipeline.

For some cancer locations, such as the prostate or head and neck, the presence of metallic implants (dental fillings and orthopedic prostheses) leads to artifacts in CT images. As the attenuation correction is required in PET/CT systems for PET quantification, the accuracy of the PET radiomic features could be degraded by CT artifacts due to metallic implants. It is therefore of interest to identify PET radiomic features robust to the presence of metallic implants.

Furthermore, automatic algorithms for lesion segmentation on PET images are preferable in order to minimize the inter- and intra-user variability and their invested time. However, the simplicity and velocity of PET segmentation algorithms are usually in expenses of a loss in segmentation accuracy, especially for heterogeneous lesions [6, 14]. Any loss in the segmentation accuracy implied by simple automatic PET segmentation approaches could translate into PET radiomic features not being reliable enough for an accurate quantification of lesion heterogeneity. Consequently, the reliability of heterogeneity quantification by PET segmentation approaches should be proved before translation into clinical use.

In this study, we firstly proposed a simple procedure to retrospectively identify those PET radiomic features robust enough to the heterogeneous protocols conveyed in a multicenter-multivendor patient cohort. We also proposed a reproducible procedure to identify PET radiomic features robust to CT metallic artifacts due to implants. In addition, we developed homemade heterogeneous phantoms to evaluate the segmentation accuracy and radiomic feature reliability given by two simple commonly used segmentation approaches.

## Materials and methods

### PET/CT imaging

PET/CT one-bed acquisition protocols were performed on three different systems (all from Philips Healthcare/Philips Medical Systems B.V, The Netherlands): GEMINI TF TOF 64 (TF64), GEMINI TF 16 Big Bore (BB), and Vereos (V). The first two systems (TF64 and BB) employed analog detectors, which is the conventionally and more commonly used design. Vereos system has a novel signal readout design based on digital detectors.

All scanners fulfilled the requirements indicated in the European Association of Nuclear Medicine (EANM) imaging guidelines and obtained the EANM Research Ltd. (EARL) accreditation during acquisition. The transverse spatial resolutions at 1 cm from the central axis of the scanner were 4.8 mm for TF64 and BB [28] and 4.2 mm for V [23] machines. PET data were corrected for random coincidences as well as for scatter and attenuation, based on the corresponding CT dataset. For TF64 and BB, the reconstruction methods for all scanners were a LOR-based ordered-subset iterative time-of-flight algorithm using spherical coordinates (BLOB) with three iterations, 33 subsets, and 0.35 relaxation parameter for smoothing. For V, 3 iterations with 9 subsets without smoothing and resolution recovery were used.

An additional attenuation correction was applied for PET images involved in the evaluation of CT artifacts on PET quantification. This attenuation correction was based on the use of the *metal artifact reduction for orthopedic implant* (OMAR) reconstruction algorithm. It is a commercial product available from Philips Healthcare, which implements an algorithm to mitigate artifacts caused by metal objects in CT images. For better comparison, all PET images were normalized by background noise, so that the mean phantom background SUV was equal to 1 [2].

### Segmentation

Different segmentation approaches were employed as follows:

- *CT threshold*. Properties of alginate, i.e., direct correspondence between alginate and the distribution of the radiotracer, permitted to define the ground truth contour of the lesion by a Hounsfield units (HU) threshold on the corresponding CT. Concretely, we employed the region-growing algorithm provided by the Medical Imaging Interaction Toolkit (MITK) 2016.11 [33], after CT image denoising with a Gaussian filter (radius = 2 voxels) and an initialization window of 50–150 HU.
- *PET 40% threshold*. A fixed threshold of 40% of the maximum intensity within the simulated lesion [3].
- *PET contrast-oriented algorithm (COA)*. An adaptive threshold taking into account the contrast between tumor concentration (mean value for a 70% isocontour of maximum intensity within the simulated lesion) and background (automatically derived from the whole image). This segmentation algorithm was previously validated by experimental phantoms and lung cancer patients [6].

### Experimental phantom measurements

Our study was divided into three main analyses. For each analysis, a different setup was employed.

#### EARL accreditation measurements with NEMA-phantom

EARL accreditation is a protocol developed by the EANM in order to ensure comparable scanner performances across multiple sites. Once the PET system is accredited, the most commonly used SUV parameters (first-order statistics) could be compared, combined, and exchanged. The accreditation protocol involves imaging of the *National Electrical Manufacturers Association (NEMA) NU 2-2012 Image Quality Phantom* (NEMA-Phantom). In multicenter trials involving PET/CT imaging, this accreditation is usually considered as a prerequisite. In our study, we proposed an extended analysis in order to additionally ensure the comparability of more complex radiomic features, i.e., not only the first-order statistics but also second- or higher-order statistics (texture features).

**Fig. 1:**
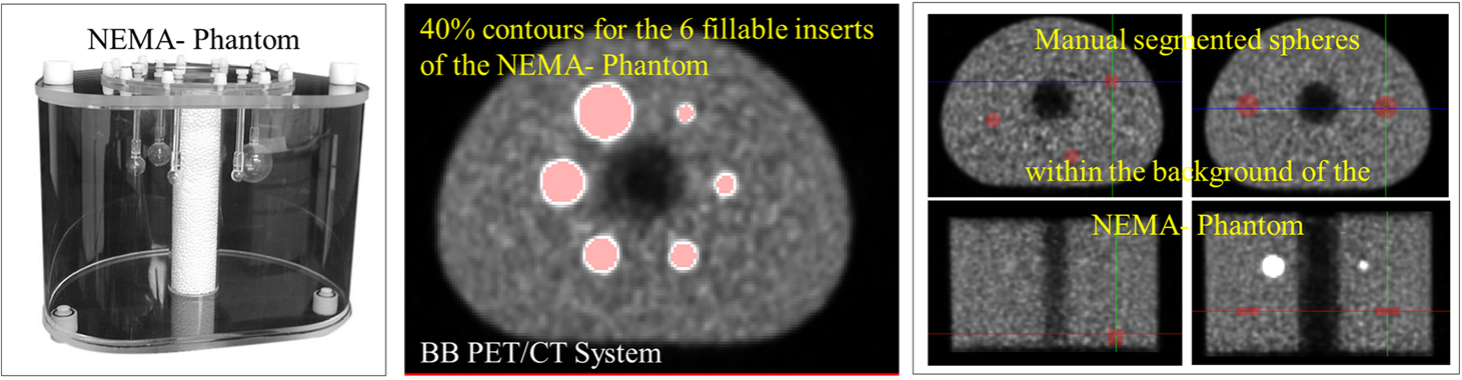
Setup employed for the identification of radiomic features robust to system and reconstruction protocol. PET images derived from the EARL accreditation measurements of NEMA-Phantom

In this section, we focused on the impact of different systems and different voxel sizes on radiomic feature variability. On each PET image, 10 spheres (5.7–8.4 cc) were manually segmented within the background of NEMA-Phantom, and the 6 fillable spheres (0.5–25 cc) were segmented by a 40% threshold (Fig. 1). First, the two analog, TF64 and BB, and one digital, V, PET/CT systems were compared. Then, the same reconstruction protocol with different voxel size, 2 × 2 × 2 mm^3^ against 4 × 4 × 4mm^3^, was compared for the BB.

#### Cheese-phantom

**Fig. 2:**
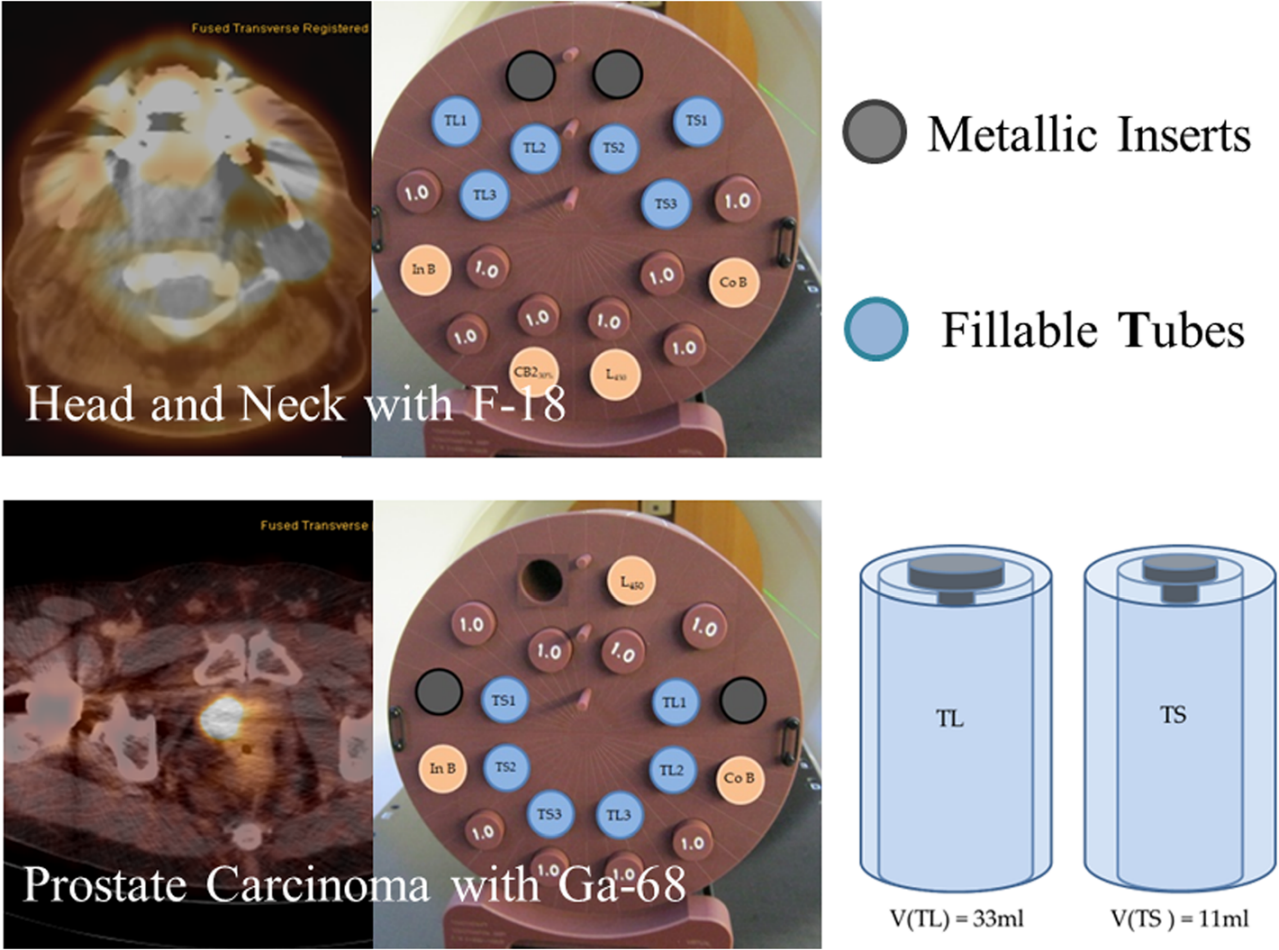
Setup employed for the identification of radiomic features robust to metal artifacts in PET/CT. Metallic (gray), tissue (brown), and fillable (large tubes (TL) and small tubes (TS), blue) inserts were placed in Cheese-Phantom following the head and neck and prostate carcinoma configurations

TomoTherapy Cheese-Phantom (Gammex RMI, Middleton, WI, USA), or equivalent phantoms from other commercial companies, are phantoms for the calibration of CT scanners available at most of the radiotherapy departments. Concretely, the Cheese-Phantom (CP) consists of an 18-cm-thick solid water cylinder with a diameter of 30 cm. The cylinder presents 20 holes with 28 mm of diameter, in which inserts representing the range of densities observed in the clinical environment (tissue inserts) could be placed (Table 1). In our study, for the evaluation of the impact of CT artifacts, three different materials (aluminum, titanium, and steel) were considered. For the simulation of the PET lesions, 6 fillable inserts were developed with a transparent material of density 1.2 g/cm^3^: 3 large tubes (TL) and 3 small tubes (TS) with volumes 33 and 11 ml, respectively. Metallic, fillable, and tissue inserts were placed in Cheese-Phantom following two configurations: head and neck and prostate carcinoma (Fig. 2). The 6 homemade inserts were filled with gallium-68 for the PC configuration and with fluorine-18 fluorodeoxyglucose (^18^F-FDG) for the head and neck configuration. The choice for the activity concentrations, volumes, and location of the fillable inserts was based on a previous quantification study of 21 prostate and 16 head and neck PET lesions. In this section, the PET/CT system employed for the 10-min scans was BB, and the segmentation of the 6 fillable spheres on PET images was performed with the 40% algorithm.

**Table 1:** Densities of the commercially available (tissue and metallic) placed on the Cheese-Phantom

Material of the insert	Density (g/cm^3^)
Lung LN-450	0.480
Solid water	1.000
Inner bone	1.136
CB2-30%	1.332
Cortical bone	1.882
Metallic inserts	
Aluminum	2.800
Titanium	4.500
Stainless steel	7.700

#### Experimental heterogeneous simulated lesions

Twenty-seven heterogeneous simulated lesions, based on a mixture of FDG and alginate [6, 7, 17], were placed in the NEMA-Phantom filled with a background concentration (C_Bg_). FDG concentrations for lesions and background were within the range reported by a previous publication in lung cancer patients [5].

**Fig. 3:**
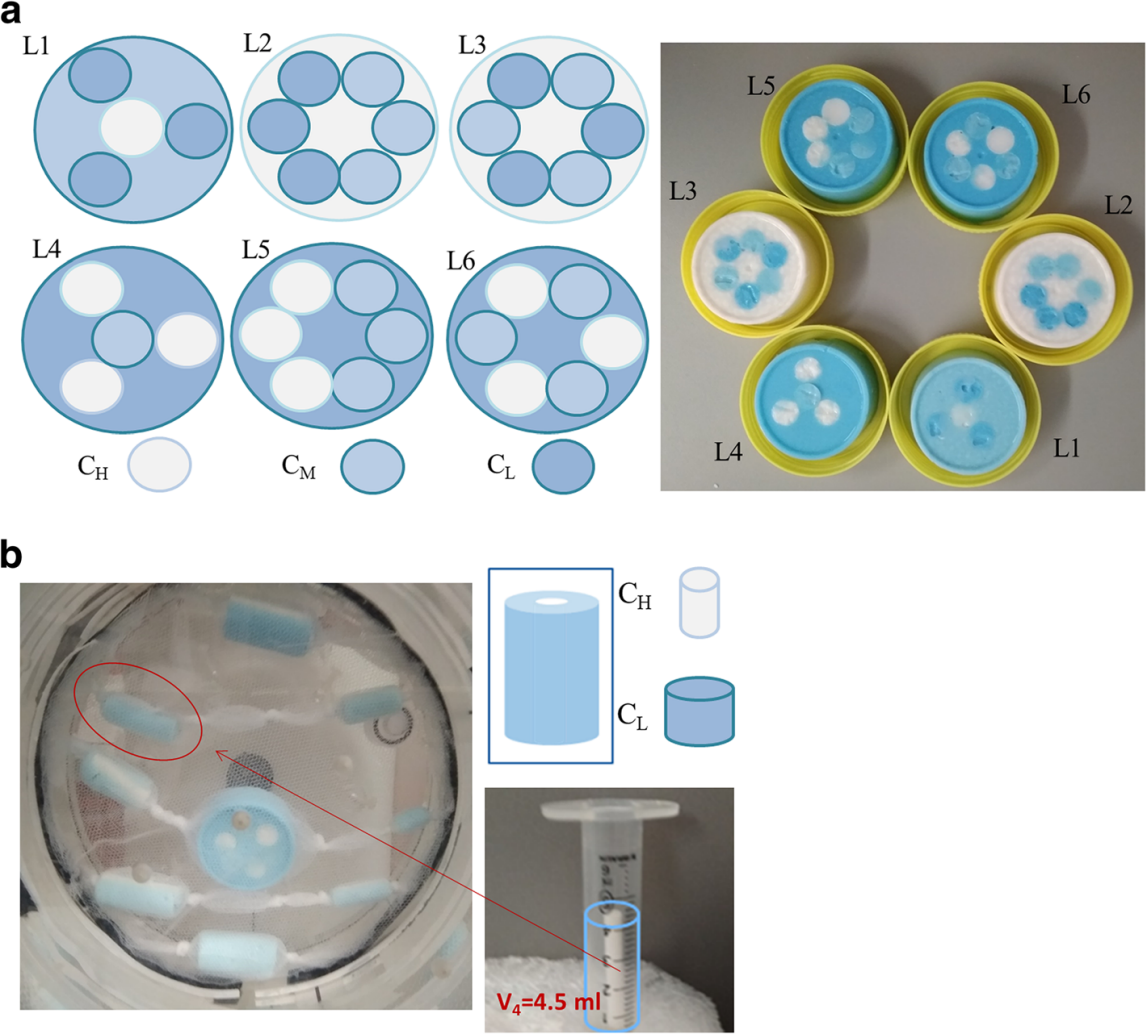
Experimental heterogeneous simulated lesions with cylindrical areas of different activity concentrations developed for the evaluation of the segmentation accuracy and the radiomic feature reliability given by automatic PET segmentation approaches. **a** Eighteen simulated lesions with a diameter longer than 3× FWHM. **b** Eight cylindrical lesions (V1 to V8) with 2 concentration layers (external cylinder with CL and inner cylinder with CH, CH/CL = 6)

The simulated lesions could be divided into two groups. In the first group, all the 18 simulated lesions had a diameter longer than 3× full width half maximum (FWHM) in order to minimize the partial volume effect (PVE) [25]. For the development of these 18 lesions, we consider six different spatial distributions within the lesions (L1–L6 in Fig. 3a) with 3 different ^18^F-FDG high (C_H_), medium (C_M_), and low (C_L_) concentrations. For each of these 6 spatial distributions, the following 3 ratios between the concentration layers within the lesion were considered: (C_H_/C_L_ = 10, C_M_/C_L_ = 5), (C_H_/C_L_ = 8, C_M_/C_L_ = 4), and (C_H_/C_L_ = 4, C_M_/C_L_ = 2). For the second group, lesions 6 (L6 in Fig. 3a) and 8 additionally developed cylindrical lesions (V1 to V8) were employed (Fig. 3b). Each cylindrical lesion had 2 concentration layers (external cylinder with C_L_ and inner cylinder with C_H_), see Fig. 3b. From V1 to V8, the volumes of both cylinders (internal and external) were progressively increased resulting in lesion volumes ranging from V1 = 1 ml to V8 = 15.6 ml. The smaller lesion would be affected by the PVE. The contrast within the lesion was C_H_/C_L_ = 6. For both groups, the contrast between the lower concentration layer and the background remained constant C_L_/C_Bg_ = 10. In this section, 10-min scans were performed with the BB PET/CT system. Three different segmentations were used: ground truth on CT, 40%, and COA.

### Feature extraction

A total of 133 features were computed with an open-source code [30] based on MATLAB® (The MathWorks Inc., Natick, MA, USA). For the radiomic feature computation, SUV values of the voxels within the contour were discretized with a fixed bin width (W = 0.01), according to the recommendation from previous publications [8, 19] and with a fixed number of bins of 64 (Nbisn = 64). Texture features were derived from four matrices: the 3D version of the gray-level co-occurrence matrix (GLCM), the gray-level run length matrix (GLRLM), the gray-level size zone matrix (GLSZM), and the neighborhood gray tone difference matrix (NGTDM). In addition, on the voxel intensities within the contour, we applied:(i) a wavelet band-pass filtering (WF), with a weight ratio of 1:2 between band-pass sub-bands and other sub-bands and (ii) an equal probability quantization algorithm (Q), by using the histeq function of MATLAB®. The radiomic features used in this study are listed in SM Table 1 of supplementary material.

### Statistical analysis

**Fig. 4:**
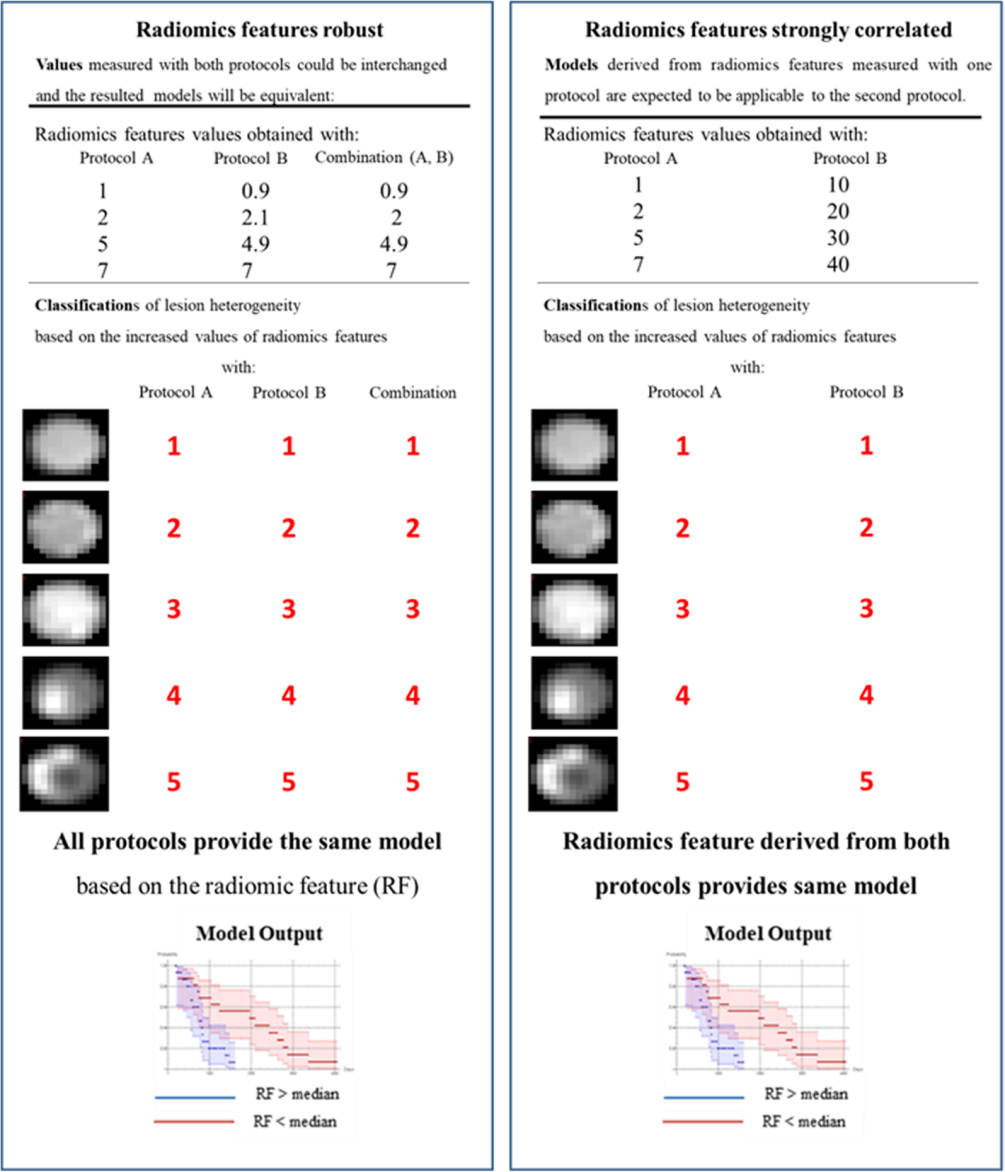
Example of radiomic features robust to different protocols (left) and radiomic features strongly correlated (right). In our study the different protocols (A and B) were PET/CT system (analogic vs digital), voxel size of reconstruction (8 mm^3^ vs 64 mm^3^), CT metal artifact (with vs without), and PET segmentation approach (ground truth vs 40% vs COA)

The statistical analysis was performed using an in-house software based on Wolfram Mathematica v 11.2. The Bonferroni correction method was applied to correct for multiple test comparisons: the significance level was lowered to a value p < α/K, where K is the number of comparisons and α is the significance level set to 0.05. When comparing two data samples, robustness was defined by the Wilcoxon signed rank test and bias quantified by Bland–Altman analysis [12]. The criterion for the significant difference was p > 0.05 for the Wilcoxon signed rank test and the mean of differences relative to the mean and its 95% confidence interval (CI) for Bland–Altman analysis. In the Bland–Altman analysis, the mean difference of comparable data sets trend to null and the 95% CI must comprise zero. Correlations were analyzed in terms of Spearman’s correlation tests, and a strong correlation was identified by p < 0.05/K (Bonferroni correction mentioned above) and r > 0.8. We were interested in the radiomic features which met one of the following criteria (Fig. 4):

(i) Radiomic features were robust, under the assumption that they will be interchangeable, and models could be therefore developed employing radiomic features derived from both protocols
(ii) Radiomic features showed a strong correlation, under the assumption that they lead to the same heterogeneity lesion classification, and therefore, they lead to equivalent radiomics models.

## Results

### Procedure for retrospective identification of radiomic features robust to different systems and reconstruction protocols

The main results are presented in Table 2. There were 48 radiomic features considered robust (Wilcoxon signed rank test) to all the different PET/CT systems, while 69 features showed significant strong correlations (r > 0.8, p < 0.05/133).

**Table 2:**
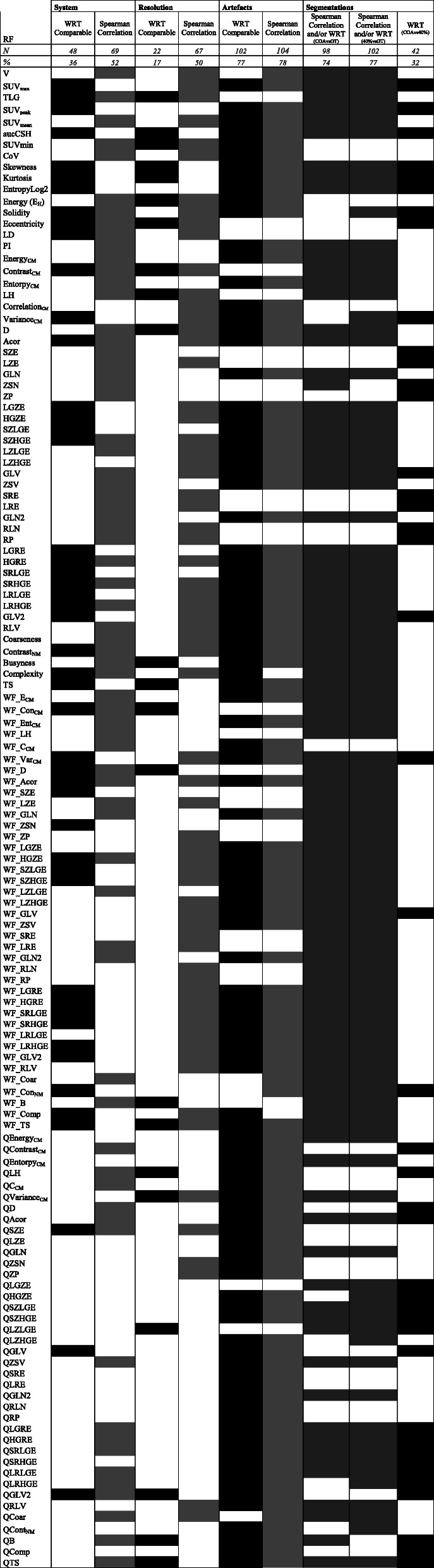
Results of RF analysis. Filled box means positive result for the analysis described on the first row (black is comparable, and gray is strongly correlated) and represents the property of interest, like for example, RF robust to the different PET/CT systems (second column)

In addition, 22 radiomic features were robust to the variation in the voxel size of the reconstruction, and 67 showed significant strong correlations. The results are provided separately for the comparisons of the system (BB vs V and TF64 vs V) (Table 2 of supplementary material). Based on the Wilcoxon signed rank test, the simple first-order radiomic features commonly used in clinical routine (SUV_mean_ and volume) were not interchangeable. Consequently, these parameters should not be recommended in studies involving different reconstruction protocols. However, they showed a strong linear correlation, and we expect therefore that a model based on SUV_mean_ and/or volume derived from a patient cohort involving 2 mm reconstruction protocol could be applied on a second cohort with a reconstruction protocol of 4 mm.

In SM Table 3 of supplementary material, we additionally presented the variability of the radiomic features computed after a discretization method with a fixed number of bins (Nbins = 64). The impact of voxel resolution resulted in a similar number of robust radiomics, but the number of linear correlations significantly decreased for Nbins = 64. The impact of the system resulted in more robust features for Nbins = 64, but a lower number of features strongly correlated.

### Reproducible procedure for identification of radiomic features robust to metallic artifacts

**Fig. 5:**
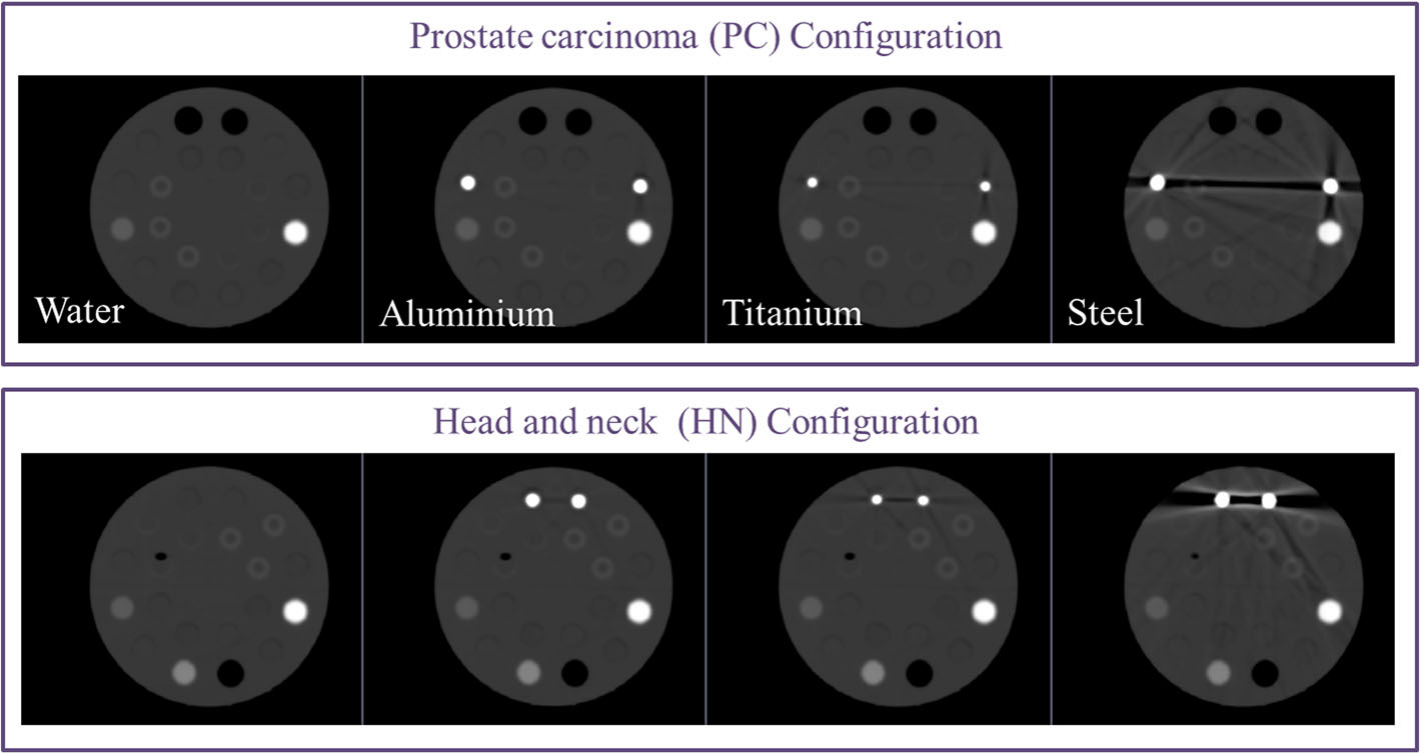
CT attenuation maps for the two prostate and head and neck Cheese-Phantom configurations

In the evaluation of CT artifacts, a reference image for each configuration was established. The reference image resulted from imaging of the Cheese-Phantom where metallic inserts were replaced by water equivalent solid water insert (Fig. 5).

On CT images, the presence of metallic inserts generated straight artifacts giving rise to an inaccurate estimation of densities. Metallic inserts were also a degrading factor for CT attenuation map image quality. The impact depended on the density of the insert, spatial distribution of the sources of artifacts, and the size of metallic inserts (Fig. 5).

For the evaluation of the robustness of PET radiomic features with respect to CT artifacts, the fillable inserts were segmented on the PET images by the 40% algorithm, and PET radiomic features were computed. We compared PET radiomic features for Cheese-Phantom with and without metallic inserts. Most radiomic features were robust to the presence of metallic elements: 125 for aluminum, 106 for titanium, and 108 for steel (Table 2 of supplementary material). Overall, 102 radiomic features were comparable for all three metals with respect to water (water insert instead metal insert), and 104 showed a strong linear correlation (Table 2). A similar reproducibility pattern was observed for Nbins = 64.

**Fig. 6:**
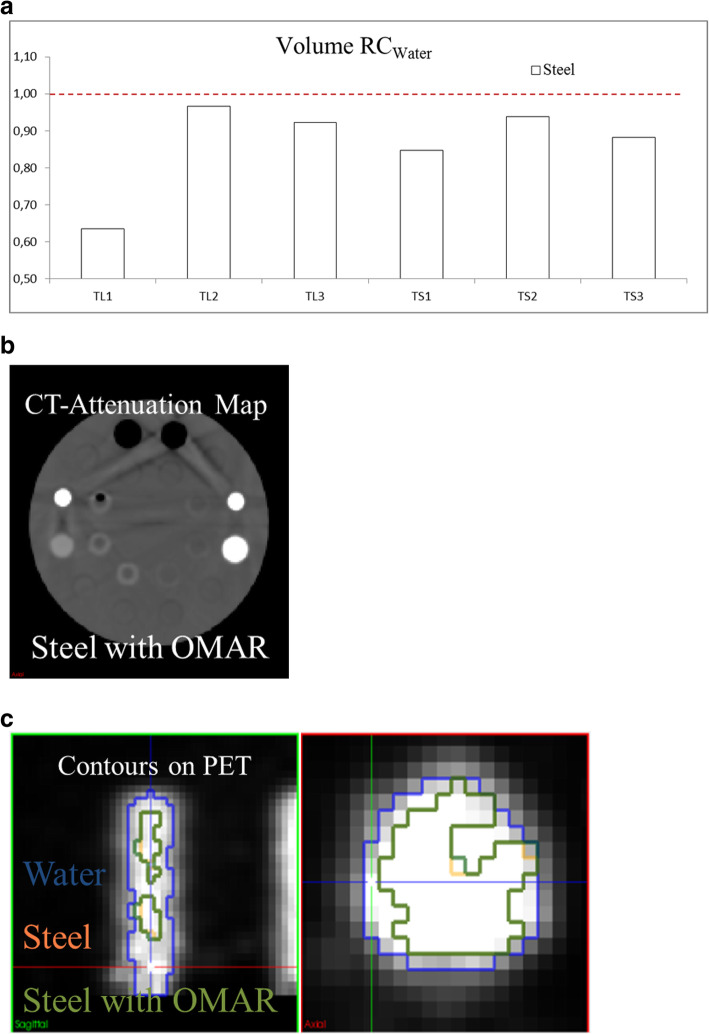
Cheese-Phantom in prostate configuration with steel inserts. **a** Recovery coefficients with respect to water (RC_water_) for the radiotracer fillable tubes. **b** CT attenuation maps and **c** PET contours for tube TL1

For the fillable inserts segmented on PET images, the volume recovery coefficients with respect to water (RC_Water_) were assessed. A significant volume underestimation was observed for the two configurations (prostate and head and neck) with steel. The largest volume underestimations were observed in the fillable inserts placed closest to the metallic inserts: TL1 (RC_Water_ = 0.64) and TS1 (RC_Water_ = 0.85) for PC configuration and TL2 (RC_Water_ = 0.82) and TS2 (RC_Water_ = 0.82) for HN configuration. In Fig. 6a, RC_Water_ for the 6 fillable inserts in PC configuration with steel are shown: average 0.87 ± 0.11, against 1.00 ± 0.08 for aluminum and 0.96 ± 0.11 for titanium. OMAR reconstruction was applied for the PC configuration, with the most significant volume underestimation (TL1 in the presence of steel insert). Although no improvement in metal density estimation was obtained, a reduction of straight artifacts was observed on both CT and CT attenuation maps (Fig. 6b). Unfortunately, this artifact reduction did not translate into an improvement neither in segmentation accuracy (Fig. 6c) nor in radiomic feature robustness: 119 were robust with OMAR and 116 without OMAR.

### Evaluation of segmentation accuracy and radiomic feature reliability for simple segmentation approaches

**Fig. 7:**
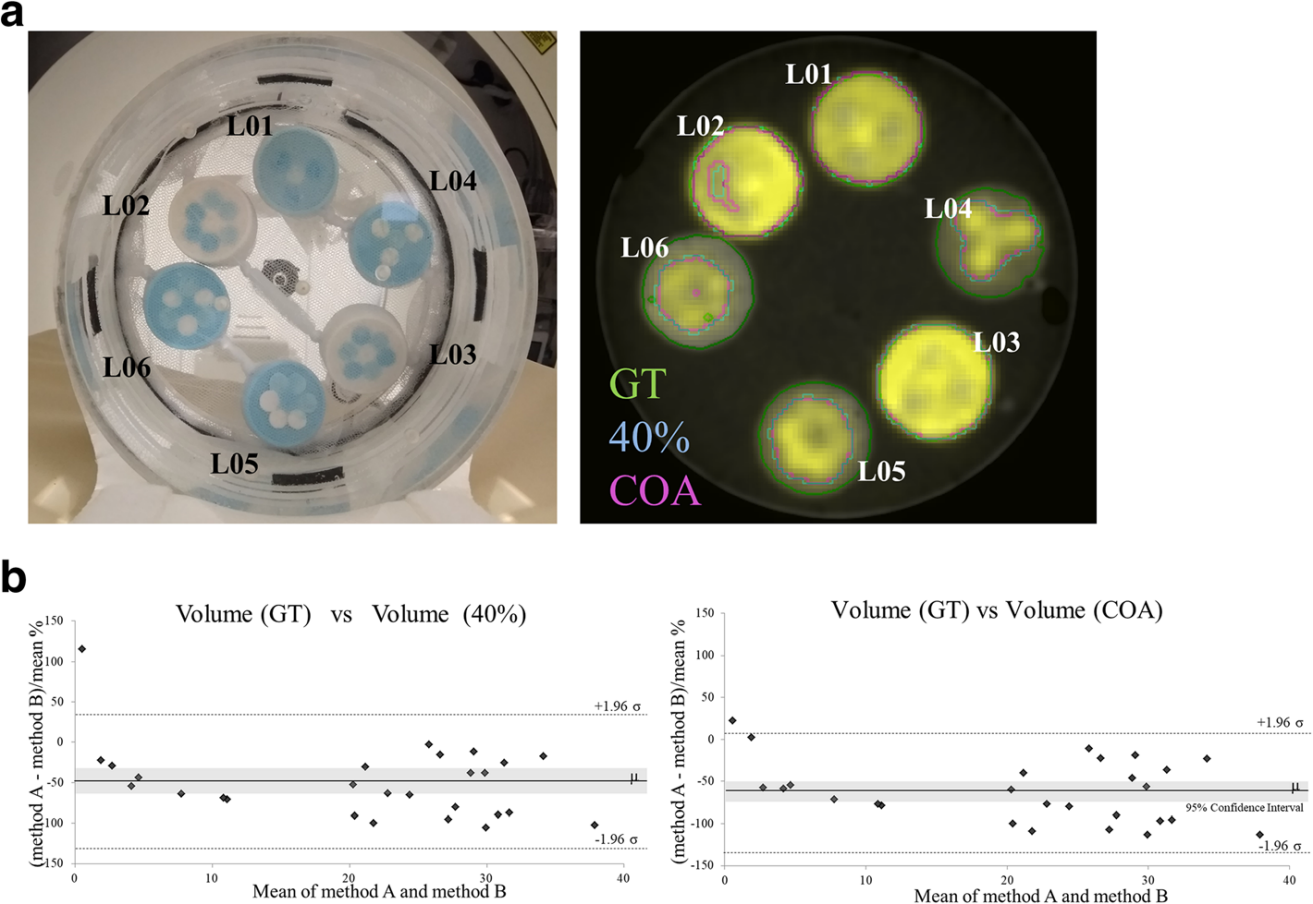
Heterogeneous simulated lesions. **a** Images and contours resulted from the 3 segmentation methods: a density threshold in the corresponding CT (ground truth), the 40% threshold PET segmentation approach (40%), and the contrast-oriented algorithm (COA). **b** Bland–Altman plot analysis for volume estimation by 40% and by COA with respect to volume estimated by GT

The threshold segmentation on CT defined the ground truth for each of the 27 heterogeneous phantoms. On PET images, two segmentation approaches (40% and COA) were applied. Overall, similar contours were observed between 40% and COA, and both of them significantly underestimated the volume with respect to the ground truth for some heterogeneous lesions. Quantification of the underestimation of volume is shown in the Bland–Altman plot analysis (Fig. 7b) for COA and 40% with respect to the ground truth. Contours for the 6 heterogeneous phantoms with concentrations C_H_/C_L_ = 8 and C_M_/C_L_ = 4 (Fig. 7a) showed that for the heterogeneous lesions having high concentrations in inner regions (L04, L05, and L06), the peripheral voxels with low concentration were erroneously rejected and labeled as non-tumor by 40% and COA.

The 42 radiomic features comparable between 40% and COA segmentations are listed in the last column of Table 2. Furthermore, despite the abovementioned significant underestimation of lesion volume, PET radiomic features derived from the ground truth contours were strongly correlated and/or comparable for 98 derived radiomic features from COA% and to 102 derived from 40%.

### Radiomic features robust to all degrading factors

From all radiomic features, only 3 histogram parameters and 1 second-order texture feature were simultaneously robust (Wilcoxon rank test) to all degrading factors: system, reconstruction voxel, artifact, and PET segmentation approach. These robust radiomic features were area under the curve of the cumulative SUV volume histogram (aucCSH), skewness (S), kurtosis (K), and gray-level variance derived from GLRLM after application of an equal probability quantization algorithm on the voxels within lesion (QGLV2). These 4 radiomic features did not correlate among themselves.

## Discussion

Fifty-five simulated lesions were employed to identify the most robust PET radiomic features with respect to the design of the PET/CT system (BB, TF64, and V), the size of the reconstructed isotropic voxel (2 mm and 4 mm), the presence of metal artifacts (aluminum, titanium, steel), and the segmentation method (40% and COA). Consequently, in studies with the abovementioned differences in acquisition, protocols, and processing, the results presented in Table 2 could be used to identify the radiomic features that are not robust enough and therefore should not be included in clinical assessments. In the following, we discuss in detail the novelty of our methodology and the significance of our results.

In our study, two of the advantages implied by the use of experimental phantoms were the possibility of comparing PET radiomics with and without the presence of metal inserts and the availability of a ground truth for the segmentation of the lesions. Previous publications evaluated the impact of segmentation methods in clinical data [15, 20]; Altazi et al. 2017; [27], but only a few publications employed experimental phantoms [1, 26]. The phantoms employed in these publications were homogeneous and had crystal walls. These two characteristics of the phantoms differed from the clinical scenario and facilitated the segmentation of the lesions. From our knowledge, apart from the three heterogeneous phantoms evaluated in a previous publication [21], we presented the first evaluation of radiomic variability with a large number of homogeneous and heterogeneous phantoms, 28 and 27, respectively. In addition, for a better simulation of the clinical case, only phantoms without walls were employed for the evaluation of the segmentation method.

Our results were in agreement with previous publications. In cervical cancer [Altazi et al. 2017], RP, SZE, and LRE showed a high level of robustness to the chosen segmentation method. For the heterogeneous phantoms in our study, these radiomic features were also comparable between segmentation methods (last column in Table 2). In addition, also in a previous evaluation with 25 patients and the 6 fillable spheres of the NEMA phantom [26], the variation of the size of the reconstruction voxel showed the most significant effect on radiomic feature variability. Furthermore, in agreement with our results, a previous investigation [15] reported a non-statistically significant difference concerning the associated predictive value of radiomic features, which had shown a high absolute difference between values derived from different delineation approaches. As expected based on previous results [6, 14], 40% and COA significantly underestimated the volume of the lesions in our study; however, most of their radiomic features showed a strong correlation and/or were comparable with respect to the radiomic features derived from the ground truth. For these radiomic features, we can therefore expect that models derived from 40% and COA segmentations will be equivalent to the model expected if the true contour of the lesion was available. This implies an important advantage for the use of radiomics in clinical routine, because both segmentation approaches are simple and easy to implement. In addition, percentage threshold segmentation is available in almost all image contouring software, including open-source tools, such as IMTK and 3D-Slicer [11], and in commercial software, such as the Eclipse treatment planning software (Varian Medical Systems, Palo Alto, CA, USA).

In the comparison of different scanners, a high-resolution PET/CT system [23] with a novel digital detector design (Vereos from Philips Healthcare) was included. Although recent publications have reported the improvement in lesion detectability conveyed by PET/CT systems with digital detectors, their impact on the robustness of the radiomic features has not been investigated previously.

Because partial volume effects compromise heterogeneous radiotracer distribution quantification, a minimum lesion size has been suggested as a requisite for quantification of intratumoral heterogeneity [4, 13]; however, there is still a lack of consensus about the limiting size to be considered. In order to cover different cohort scenarios, i.e., different range of volumes considered for the radiomics analysis, the robustness of the radiomic features was analyzed separately for two groups: (i) 18 large lesions (diameter longer than 3× FWHM) and (ii) 9 lesions with volume ranging from 1 to 16 ml (Table 2 of supplementary material). Consequently, for a given PET radiomics analysis and applied lesion size criteria, we recommend referring to the results of our study reported for the group of simulated lesions with a range of volumes more similar to the range of volumes involved in the evaluation.

Previous publications [[19]; van Helden et al. 2016; [22] have investigated the impact of the discretization method on the radiomic feature variability. In agreement with these publications, our group also recommended the discretization method with a fixed width [8]. Consequently, in the current study, our analysis focused on the radiomic features computed after discretization with a fixed SUV width of 0.01. However, in SM Table 3 of electronic supplementary material, we additionally presented the variability observed for radiomic features after a discretization with a fixed number of bins (Nbins = 64). The results confirmed that different discretization methods yielded different reproducibility of the image features.

Our results confirm a non-negligible variability of radiomic features due to different factors. The standardization of acquisition and post-processing protocols has been recommended in order to minimize this variability. The posteriori harmonization of the data derived from different protocols has been also suggested as an alternative approach [9]. Harmonization has been employed in other imaging modalities such as magnetic resonance, but it is still under investigation in PET. In this work, we proposed the use of EARL accreditation to retrospectively reject the radiomic features which are not robust enough. It could be of special interest in studies involving patients from different institutions or patients that underwent PET/CT acquisitions with systems which are no longer in use at the department. In addition, in the evaluation of the impact of CT artifacts on radiomic feature variability, the reproducibility of the proposed procedure relied on the availability of the Cheese-Phantom at most radiotherapy departments. Consequently, not only our results could be employed by other institutions, also the procedures proposed could be used for the evaluation of additional degrading factors in large heterogeneous series of patients.

Furthermore, the use of experimental phantoms has the benefit of allowing a retrospective and reproducible analysis and ensuring that the variability observed in the radiomic features is mainly due to image properties. However, the main limitation of this study is that, as suggested in previous publications [10, 18, 29, 31], variability due to non-stable physiologic processes should be also considered to reject radiomic features not stable enough for a radiomics analysis. Our study provided PET radiomic features that should be rejected. However, before using the remaining PET radiomic features, a further analysis focused on the evaluation of their stability for the specific cancer site and the concrete radiotracer involved in the corresponding patient cohort is recommended. A second limitation is the fact that our reported results on robustness are limited to the systems and protocols used in the study. Further analysis for the transferability of results is recommended as the presented procedures can be easily reproduced in other institutions.

## Conclusion

The robustness of PET radiomic features based on experimental phantom measurements has been provided. A reproducible methodology to retrospectively identify radiomic features robust to different factors with impact on their variability showed that 4 radiomic features were robust to all degrading factors and, therefore, of special interest for PET quantification: area under the curve of the cumulative SUV volume histogram (aucCSH), skewness (S), kurtosis (K), and gray-level variance derived from GLRLM after application of an equal-probability quantization algorithm on the voxels within lesion (QGLV2). In addition, our results supported the use of 40% and COA segmentation approaches in radiomics analysis.

## Supplementary Information

**Additional file 1: Table S1.** Radiomics Features. Abbreviations: SUV: standardized uptake values, GLCM: gray-level co-occurrence matrix, GLRLM: gray-level run length matrix, GLSZM: gray-level size zone matrix, NGTDM: neighborhood gray tone difference matrix. Parameters derived from GLCM, GLSZM, GLRLM and NGTDM (texture features) were also computed after applying (i) a wavelet band-pass filtering (prefix WF-), with a weight ratio 1:2 between band-pass sub-bands and other sub-bands and (ii) an equal-probability quantization algorithm (prefix Q-) on the voxel intensities within the contour.

**Additional file 2: Table S2.** Detailed results of RF analysis. Filled box means positive result for the analysis described on the first row (black is comparable and gray is strong correlated) and represents the property of interest; like for example, RF robust to the different PET/CT systems (second column).

**Additional file 3: Table S3.** Results of RF analysis for a resampling pre-processing with a constant number of bins N=64. Filled box means positive result for the analysis described on the first row (black is comparable and gray is strong correlated) and represents the property of interest; like for example, RF robust to the different PET/CT systems (second column).

## Data Availability

The datasets during and/or analyzed during the current study are available from the corresponding author on reasonable request.

## Abbreviations

PET: Positron emission tomography
CT: Computed tomography
SUV: Standardized uptake value
EANM: European Association of Nuclear Medicine
EARL: European Association Research Ltd.
NEMA: National Electrical Manufacturers Association
VOI: Volume of interest
FDG: Fluorine-18 fluorodeoxyglucose
HU: Hounsfield units
