# Supplementary material for: Experimental phantom evaluation to identify robust positron emission tomography (PET) radiomic features
Source: EJNMMI Phys. 2021 Jun 12;8:46. doi: 10.1186/s40658-021-00390-7 (PMC8197692; doi:10.1186/s40658-021-00390-7)
Supplement: Supplementary file 1 — Additional file 1: Table S1. Radiomics Features. Abbreviations: SUV: standardized uptake values, GLCM: gray-level co-occurrence matrix, GLRLM: gray-level run length matrix, GLSZM: gray-level size zone matrix, NGTDM: neighborhood gray tone difference matrix. Parameters derived from GLCM, GLSZM, GLRLM and NGTDM (texture features) were also computed after applying (i) a wavelet band-pass filtering (prefix WF-), with a weight ratio 1:2 between band-pass sub-bands and other sub-bands and (ii) an equal-probability quantization algorithm (prefix Q-) on the voxel intensities within the contour. [file 40658_2021_390_MOESM1_ESM.docx]

| SUV-Histogram | Maximum SUV (SUV_max_), Peak SUV (SUV_peak_), Minimum SUV (SUV_max_), |
| --- | --- |
|  | Mean SUV (SUV_mean_), Volume (V), Total Lesion Glicolisis (TLG),  Area under the curve of the cumulative SUV-volume histogram (aucCSH),  Skewness (S), Coefficient of variance (CoV), Kurtosis (K),  Energy(E_H_) and Entropy (Ent_H_), |
| Geometry | Solidity (S), Eccentricity (Ecc ), Long Diameter (LD), Percent Inactive (PI) |
| GLCM | Local Homogeneity (LH), Correlation (C_CM_ ), Contrast (Con_CM_), |
|  | Energy (E_CM_), Entropy (Ent_CM_), Variance (Var_CM_ ),  Autocorrelation (Acor ) and Dissimilarity (D) |
| GLSZM | Small Zone Emphasis (SZE), Large Zone Emphasis (LZE), |
|  | Low Gray-Level Zone Emphasis (LGZE), High Gray-Level Zone Emphasis (HGZE),  Small Zone Low Gray-Level Emphasis (SZLGE), Small Zone High  Gray-Level Emphasis (SZHGE), Large Zone Low Gray-Level Emphasis(LZLGE),  Large Zone High Gray-Level Emphasis (LZHGE), |
|  | Zone-Size Non-uniformity (ZSN), Zone Percentage (ZP),  Gray-Level Variance (GLV), Gray-Level Non-uniformity (GLN2)  and Zone-Size Variance (ZSV) |
| GLRLM | Short Run Emphasis (SRE), Long Run Emphasis (LRE), |
|  | Low Gray-Level Run Emphasis (LGRE), High Gray-Level Run Emphasis (HGRE) |
|  | Short Run Low Gray-Level Emphasis (SRLGE), Short Run High Gray-Level  Emphasis (SRHGE), Long Run Low Gray-Level Emphasis (LRLGE),  Long Run High Gray-Level Emphasis (LRHGE),  Gray-Level Non-uniformity (GLN2), Gray-Level Variance (GLV2),  Run Percentage (RP) and Length Non-uniformity (RLN) |
| NGTDM | Coarseness (Coar), Contrast (Con_NM_), |
|  | Complexity (Comp) and Texture Strength (TS) |

**ESM Table 1. Radiomics Features**

Abbreviations: SUV: standardized uptake values, GLCM: gray-level co-occurrence matrix, GLRLM: gray-level run length matrix, GLSZM: gray-level size zone matrix, NGTDM: neighborhood gray tone difference matrix.

Parameters derived from GLCM, GLSZM, GLRLM and NGTDM (texture features) were also computed after applying (i) a **wavelet band-pass filtering** (prefix **WF-**), with a weight ratio 1:2 between band-pass sub-bands and other sub-bands and (ii) an **equal-probability quantization** algorithm (prefix **Q-**) on the voxel intensities within the contour.
