# Supplementary material for: Experimental phantom evaluation to identify robust positron emission tomography (PET) radiomic features
Source: EJNMMI Phys. 2021 Jun 12;8:46. doi: 10.1186/s40658-021-00390-7 (PMC8197692; doi:10.1186/s40658-021-00390-7)
Supplement: Supplementary file 2 — Additional file 2: Table S2. Detailed results of RF analysis. Filled box means positive result for the analysis described on the first row (black is comparable and gray is strong correlated) and represents the property of interest; like for example, RF robust to the different PET/CT systems (second column). [file 40658_2021_390_MOESM2_ESM.docx]

**ESM_Table2.** Detailed results of RF analysis. Filled box means positive result for the analysis described on the first row (black is comparable and gray is strong correlated) and represents the property of interest; like for example, RF robust to the different PET/CT systems (second column).

|  | **TF64 vs V** | | **BB vs V** | |  | **Aluminium**  **vs**  **Water** | | **Titanum**  **vs**  **Water** | | **Steel**  **vs**  **Water** | |  | **Segmentations (18 Lesions)** | **Segmentations**  **(9 Lesions)** |
| --- | --- | --- | --- | --- | --- | --- | --- | --- | --- | --- | --- | --- | --- | --- |
| RF | WRT | S | WRT | S | | WRT | S | WRT | S | WRT | S | | WRT (COAvs40%) | WRT (COAvs40%) |
| *N* | *80* | *104* | *55* | *75* | | *125* | *128* | *106* | *109* | *108* | *111* | | *48* | *121* |
| *%* | *60* | *78* | *41* | *56* | | *94* | *96* | *80* | *82* | *81* | *83* | | *36* | *91* |
| V | 0 | 1 | 1 | 1 | | 1 | 1 | 1 | 1 | 0 | 1 | | 0 | 0 |
| SUV_max_ | 1 | 1 | 1 |  | | 1 | 1 | 1 | 1 | 1 | 1 | | 1 | 1 |
| TLG | 1 | 1 | 1 | 1 | | 1 | 1 | 1 | 1 | 0 | 0 | | 0 | 0 |
| SUV_peak_ | 1 | 1 | 1 |  | | 1 | 1 | 1 | 1 | 1 | 1 | | 1 | 1 |
| SUV_mean_ | 0 | 1 | 1 | 1 | | 1 | 1 | 1 | 1 | 1 | 1 | | 0 | 0 |
| aucCSH | 1 |  | 1 |  | | 1 | 1 | 1 | 1 | 1 | 1 | | 1 | 1 |
| SUVmin | 0 | 1 | 0 | 1 | | 1 | 1 | 1 | 1 | 1 | 1 | | 0 | 1 |
| CoV | 0 | 1 | 0 | 1 | | 1 | 1 | 1 | 1 | 1 | 1 | | 0 | 0 |
| Skewness | 1 |  | 1 |  | | 1 | 1 | 1 | 1 | 1 | 1 | | 0 | 1 |
| Kurtosis | 1 |  | 1 |  | | 1 | 1 | 1 | 1 | 1 | 1 | | 1 | 1 |
| EntropyLog2 | 1 |  | 1 |  | | 1 | 1 | 1 | 1 | 1 | 1 | | 0 | 1 |
| Energy (E_H_) | 0 | 1 | 0 | 1 | | 1 | 1 | 1 | 1 | 1 | 1 | | 0 | 0 |
| Solidity | 1 | 1 | 1 | 1 | | 1 | 1 | 1 | 1 | 1 | 1 | | 1 | 1 |
| Eccentricity | 1 | 1 | 1 | 1 | | 0 | 0 | 0 | 0 | 0 | 0 | | 1 | 1 |
| LD | 1 | 1 | 1 | 1 | | 0 | 0 | 0 | 0 | 0 | 0 | | 0 | 0 |
| PI | 0 | 1 | 1 | 1 | | 1 | 1 | 1 | 1 | 1 | 1 | | 0 | 1 |
| Energy_CM_ | 0 | 1 | 0 | 1 | | 1 | 1 | 1 | 1 | 1 | 1 | | 0 | 1 |
| Contrast_CM_ | 1 | 1 | 1 | 1 | | 1 | 1 | 0 | 0 | 1 | 1 | | 0 | 1 |
| Entorpy_CM_ | 0 | 1 | 0 | 1 | | 1 | 1 | 1 | 1 | 1 | 1 | | 0 | 1 |
| LH | 0 | 1 | 0 | 1 | | 1 | 1 | 0 | 0 | 0 | 0 | | 0 | 1 |
| Correlation_CM_ | 0 |  | 0 |  | | 1 | 1 | 1 | 1 | 1 | 1 | | 0 | 1 |
| Variance_CM_ | 1 |  | 1 | 1 | | 1 | 1 | 1 | 1 | 1 | 1 | | 1 | 1 |
| D | 1 | 1 | 0 | 1 | | 1 | 1 | 1 | 1 | 1 | 1 | | 0 | 1 |
| Acor | 1 | 1 | 1 | 1 | | 1 | 1 | 1 | 1 | 1 | 1 | | 0 | 1 |
| SZE | 1 | 1 | 0 | 1 | | 1 | 1 | 0 | 0 | 0 | 0 | | 1 | 1 |
| LZE | 0 | 1 | 0 | 1 | | 0 | 0 | 0 | 0 | 0 | 0 | | 1 | 1 |
| GLN | 1 | 1 | 0 | 1 | | 1 | 1 | 1 | 1 | 1 | 1 | | 0 | 1 |
| ZSN | 1 | 1 | 0 | 1 | | 1 | 1 | 0 | 0 | 0 | 0 | | 1 | 1 |
| ZP | 1 | 1 | 0 | 1 | | 1 | 1 | 0 | 0 | 0 | 0 | | 1 | 1 |
| LGZE | 1 |  | 1 |  | | 1 | 1 | 1 | 1 | 1 | 1 | | 0 | 1 |
| HGZE | 1 | 1 | 1 |  | | 1 | 1 | 1 | 1 | 1 | 1 | | 0 | 1 |
| SZLGE | 1 |  | 1 |  | | 1 | 1 | 1 | 1 | 1 | 1 | | 0 | 1 |
| SZHGE | 1 | 1 | 1 | 1 | | 1 | 1 | 1 | 1 | 1 | 1 | | 0 | 1 |
| LZLGE | 0 | 1 | 0 | 1 | | 1 | 1 | 1 | 1 | 1 | 1 | | 0 | 1 |
| LZHGE | 1 |  | 0 |  | | 1 | 1 | 1 | 1 | 1 | 1 | | 0 | 1 |
| GLV | 1 | 1 | 0 | 1 | | 1 | 1 | 1 | 1 | 1 | 1 | | 1 | 1 |
| ZSV | 0 | 1 | 0 | 1 | | 1 | 1 | 1 | 1 | 1 | 1 | | 0 | 1 |
| SRE | 0 | 1 | 0 | 1 | | 1 | 1 | 0 | 1 | 0 | 0 | | 1 | 1 |
| LRE | 0 | 1 | 0 | 1 | | 1 | 1 | 0 | 1 | 0 | 0 | | 1 | 1 |
| GLN2 | 0 | 1 | 0 | 1 | | 1 | 1 | 1 | 1 | 1 | 1 | | 0 | 1 |
| RLN | 0 | 1 | 0 | 1 | | 1 | 1 | 0 | 1 | 0 | 0 | | 1 | 1 |
| RP | 0 | 1 | 0 | 1 | | 1 | 1 | 0 | 1 | 0 | 0 | | 1 | 1 |
| LGRE | 1 |  | 1 |  | | 1 | 1 | 1 | 1 | 1 | 1 | | 0 | 1 |
| HGRE | 1 | 1 | 1 | 1 | | 1 | 1 | 1 | 1 | 1 | 1 | | 0 | 1 |
| SRLGE | 1 |  | 1 |  | | 1 | 1 | 1 | 1 | 1 | 1 | | 0 | 1 |
| SRHGE | 1 | 1 | 1 | 1 | | 1 | 1 | 1 | 1 | 1 | 1 | | 0 | 1 |
| LRLGE | 1 |  | 1 |  | | 1 | 1 | 1 | 1 | 1 | 1 | | 0 | 1 |
| LRHGE | 1 | 1 | 1 | 1 | | 1 | 1 | 1 | 1 | 1 | 1 | | 0 | 1 |
| GLV2 | 1 |  | 1 |  | | 1 | 1 | 1 | 1 | 1 | 1 | | 1 | 1 |
| RLV | 0 | 1 | 0 | 1 | | 1 | 1 | 1 | 1 | 1 | 1 | | 0 | 1 |
| Coarseness | 0 | 1 | 0 | 1 | | 1 | 1 | 1 | 1 | 1 | 1 | | 0 | 1 |
| Contrast_NM_ | 1 | 1 | 1 | 1 | | 1 | 1 | 1 | 1 | 1 | 1 | | 0 | 1 |
| Busyness | 1 | 1 | 0 | 1 | | 1 | 1 | 1 | 1 | 1 | 1 | | 0 | 1 |
| Complexity | 1 | 1 | 1 | 1 | | 1 | 1 | 1 | 0 | 1 | 1 | | 0 | 0 |
| TS | 1 | 1 | 1 |  | | 1 | 1 | 1 | 1 | 1 | 1 | | 0 | 0 |
| WF_E_CM_ | 0 | 1 | 0 | 1 | | 1 | 1 | 1 | 1 | 1 | 1 | | 0 | 1 |
| WF_Con_CM_ | 1 | 1 | 1 | 1 | | 1 | 1 | 0 | 0 | 1 | 1 | | 0 | 0 |
| WF_Ent_CM_ | 0 | 1 | 0 | 1 | | 1 | 1 | 1 | 1 | 1 | 1 | | 0 | 1 |
| WF_LH | 1 | 1 | 0 | 1 | | 1 | 1 | 0 | 0 | 0 | 0 | | 0 | 0 |
| WF_C_CM_ | 0 | 1 | 0 | 1 | | 1 | 1 | 1 | 1 | 1 | 1 | | 0 | 1 |
| WF_Var_CM_ | 1 |  | 1 | 1 | | 1 | 1 | 1 | 1 | 1 | 1 | | 1 | 1 |
| WF_D | 1 | 1 | 1 | 1 | | 1 | 1 | 0 | 0 | 1 | 1 | | 0 | 1 |
| WF_Acor | 1 | 1 | 1 | 1 | | 1 | 1 | 1 | 1 | 1 | 1 | | 0 | 1 |
| WF_SZE | 1 |  | 1 |  | | 1 | 1 | 0 | 0 | 0 | 0 | | 1 | 1 |
| WF_LZE | 0 | 1 | 0 | 1 | | 0 | 0 | 0 | 0 | 0 | 0 | | 1 | 1 |
| WF_GLN | 0 | 1 | 0 | 1 | | 1 | 1 | 1 | 1 | 1 | 1 | | 0 | 1 |
| WF_ZSN | 1 |  | 1 |  | | 1 | 1 | 0 | 0 | 0 | 0 | | 1 | 1 |
| WF_ZP | 1 | 1 | 0 |  | | 0 | 0 | 0 | 0 | 0 | 0 | | 1 | 1 |
| WF_LGZE | 1 |  | 0 |  | | 1 | 1 | 1 | 1 | 1 | 1 | | 0 | 1 |
| WF_HGZE | 1 | 1 | 1 | 1 | | 1 | 1 | 1 | 1 | 1 | 1 | | 0 | 1 |
| WF_SZLGE | 1 |  | 1 |  | | 1 | 1 | 1 | 1 | 1 | 1 | | 0 | 1 |
| WF_SZHGE | 1 | 1 | 1 |  | | 1 | 1 | 1 | 1 | 1 | 1 | | 0 | 1 |
| WF_LZLGE | 1 | 1 | 0 | 1 | | 1 | 1 | 1 | 1 | 1 | 1 | | 0 | 1 |
| WF_LZHGE | 0 |  | 0 |  | | 1 | 1 | 1 | 1 | 1 | 1 | | 0 | 1 |
| WF_GLV | 1 | 1 | 0 |  | | 1 | 1 | 1 | 1 | 1 | 1 | | 1 | 1 |
| WF_ZSV | 0 | 1 | 0 |  | | 1 | 1 | 1 | 1 | 1 | 1 | | 0 | 1 |
| WF_SRE | 0 | 1 | 0 |  | | 0 | 1 | 0 | 0 | 0 | 0 | | 1 | 1 |
| WF_LRE | 0 | 1 | 0 | 1 | | 0 | 1 | 0 | 0 | 0 | 0 | | 1 | 1 |
| WF_GLN2 | 0 | 1 | 0 | 1 | | 1 | 1 | 1 | 1 | 1 | 1 | | 0 | 1 |
| WF_RLN | 1 | 1 | 0 |  | | 1 | 1 | 0 | 0 | 0 | 0 | | 1 | 1 |
| WF_RP | 0 | 1 | 0 |  | | 0 | 1 | 0 | 0 | 0 | 0 | | 1 | 1 |
| WF_LGRE | 1 |  | 1 |  | | 1 | 1 | 1 | 1 | 1 | 1 | | 0 | 1 |
| WF_HGRE | 1 |  | 1 | 1 | | 1 | 1 | 1 | 1 | 1 | 1 | | 0 | 1 |
| WF_SRLGE | 1 |  | 1 |  | | 1 | 1 | 1 | 1 | 1 | 1 | | 0 | 1 |
| WF_SRHGE | 1 |  | 1 | 1 | | 1 | 1 | 1 | 1 | 1 | 1 | | 0 | 1 |
| WF_LRLGE | 1 |  | 0 |  | | 1 | 1 | 1 | 1 | 1 | 1 | | 0 | 1 |
| WF_LRHGE | 1 |  | 1 | 1 | | 1 | 1 | 1 | 1 | 1 | 1 | | 0 | 1 |
| WF_GLV2 | 1 |  | 1 |  | | 1 | 1 | 1 | 1 | 1 | 1 | | 1 | 1 |
| WF_RLV | 0 | 1 | 0 |  | | 1 | 1 | 1 | 1 | 1 | 1 | | 0 | 1 |
| WF_Coar | 0 | 1 | 1 | 1 | | 1 | 1 | 1 | 1 | 0 | 1 | | 0 | 1 |
| WF_Con_NM_ | 1 |  | 1 |  | | 1 | 1 | 0 | 1 | 1 | 1 | | 1 | 1 |
| WF_B | 1 | 1 | 0 | 1 | | 1 | 1 | 0 | 0 | 1 | 1 | | 0 | 1 |
| WF_Comp | 1 | 1 | 1 |  | | 1 | 1 | 1 | 0 | 1 | 1 | | 0 | 0 |
| WF_TS | 1 | 1 | 1 |  | | 1 | 1 | 1 | 1 | 1 | 1 | | 0 | 0 |
| QEnergy_CM_ | 0 | 1 | 0 |  | | 1 | 1 | 1 | 1 | 1 | 1 | | 0 | 1 |
| QContrast_CM_ | 1 | 1 | 0 | 1 | | 1 | 1 | 1 | 1 | 1 | 1 | | 1 | 1 |
| QEntorpy_CM_ | 0 | 1 | 0 |  | | 1 | 1 | 1 | 1 | 1 | 1 | | 0 | 1 |
| QLH | 0 | 1 | 0 | 1 | | 1 | 1 | 1 | 1 | 1 | 1 | | 1 | 1 |
| QC_CM_ | 0 | 1 | 0 | 1 | | 1 | 1 | 1 | 1 | 1 | 1 | | 0 | 1 |
| QVariance_CM_ | 0 |  | 1 |  | | 1 | 1 | 1 | 1 | 1 | 1 | | 0 | 1 |
| QD | 1 | 1 | 0 | 1 | | 1 | 1 | 1 | 1 | 1 | 1 | | 1 | 1 |
| QAcor | 1 | 1 | 0 | 1 | | 1 | 1 | 1 | 1 | 1 | 1 | | 1 | 1 |
| QSZE | 1 | 1 | 1 | 1 | | 1 | 1 | 1 | 1 | 1 | 1 | | 0 | 1 |
| QLZE | 0 | 1 | 0 |  | | 1 | 1 | 1 | 1 | 1 | 1 | | 0 | 1 |
| QGLN | 1 | 1 | 0 |  | | 1 | 1 | 1 | 1 | 1 | 1 | | 0 | 1 |
| QZSN | 1 | 1 | 0 |  | | 1 | 1 | 1 | 1 | 1 | 1 | | 0 | 1 |
| QZP | 0 | 1 | 0 |  | | 1 | 1 | 1 | 1 | 1 | 1 | | 0 | 1 |
| QLGZE | 0 | 1 | 0 |  | | 1 | 1 | 0 | 0 | 0 | 0 | | 1 | 1 |
| QHGZE | 1 | 1 | 0 |  | | 1 | 1 | 1 | 1 | 1 | 1 | | 1 | 1 |
| QSZLGE | 0 | 1 | 0 |  | | 1 | 1 | 1 | 1 | 1 | 1 | | 1 | 1 |
| QSZHGE | 1 | 1 | 0 |  | | 1 | 1 | 1 | 1 | 1 | 1 | | 1 | 1 |
| QLZLGE | 1 |  | 0 | 1 | | 1 | 1 | 0 | 0 | 1 | 1 | | 1 | 1 |
| QLZHGE | 0 | 1 | 1 |  | | 1 | 1 | 1 | 1 | 1 | 1 | | 0 | 1 |
| QGLV | 1 | 1 | 1 |  | | 1 | 1 | 1 | 1 | 1 | 1 | | 0 | 1 |
| QZSV | 0 | 1 | 0 | 1 | | 1 | 1 | 1 | 1 | 1 | 1 | | 0 | 1 |
| QSRE | 0 | 1 | 0 |  | | 1 | 1 | 1 | 1 | 1 | 1 | | 0 | 1 |
| QLRE | 1 | 1 | 0 |  | | 1 | 1 | 1 | 1 | 1 | 1 | | 0 | 1 |
| QGLN2 | 0 | 1 | 0 |  | | 1 | 1 | 1 | 1 | 1 | 1 | | 0 | 1 |
| QRLN | 0 | 1 | 0 |  | | 1 | 1 | 1 | 1 | 1 | 1 | | 0 | 1 |
| QRP | 1 | 1 | 0 |  | | 1 | 1 | 1 | 1 | 1 | 1 | | 0 | 1 |
| QLGRE | 0 | 1 | 0 | 1 | | 1 | 1 | 1 | 1 | 1 | 1 | | 1 | 1 |
| QHGRE | 1 | 1 | 0 | 1 | | 1 | 1 | 1 | 1 | 1 | 1 | | 1 | 1 |
| QSRLGE | 0 | 1 | 0 | 1 | | 1 | 1 | 1 | 1 | 1 | 1 | | 1 | 1 |
| QSRHGE | 1 | 1 | 0 |  | | 1 | 1 | 1 | 1 | 1 | 1 | | 1 | 1 |
| QLRLGE | 0 | 1 | 0 | 1 | | 1 | 1 | 1 | 1 | 1 | 1 | | 1 | 1 |
| QLRHGE | 1 | 1 | 0 | 1 | | 1 | 1 | 1 | 1 | 1 | 1 | | 1 | 1 |
| QGLV2 | 1 | 1 | 1 | 1 | | 1 | 1 | 1 | 1 | 1 | 1 | | 0 | 1 |
| QRLV | 0 | 1 | 0 |  | | 1 | 1 | 1 | 1 | 1 | 1 | | 0 | 1 |
| QCoar | 0 | 1 | 1 | 1 | | 1 | 1 | 1 | 1 | 0 | 1 | | 0 | 1 |
| QCont_NM_ | 1 | 1 | 0 |  | | 1 | 1 | 1 | 1 | 1 | 1 | | 1 | 1 |
| QB | 0 | 1 | 0 | 1 | | 1 | 1 | 1 | 1 | 1 | 1 | | 1 | 1 |
| QComp | 1 | 1 | 0 |  | | 1 | 1 | 1 | 1 | 1 | 1 | | 1 | 1 |
| QTS | 1 | 1 | 0 | 1 | | 1 | 1 | 1 | 1 | 1 | 1 | | 1 | 1 |
